# Current decision support tools fail to agree or predict therapeutic decisions in a single cohort of unruptured intracranial aneurysms

**DOI:** 10.1007/s00701-021-04852-w

**Published:** 2021-05-06

**Authors:** Ahilan Kailaya-Vasan, Joseph Frantzias, Jayantan Kailaya-Vasan, Ian A. Anderson, Daniel C. Walsh

**Affiliations:** 1 Department of Neurosurgery, King’s College Hospital, Denmark Hill, Brixton, London, SE5 9RS UK; 2 Institute of Psychiatry, King’s College London, DeCrespigny Park, London, UK

**Keywords:** Unruptured intracranial aneurysms, Treatment score, Multi-disciplinary meeting, Treatment, Screening

## Abstract

**Background:**

There is limited evidence to direct the management of unruptured intracranial aneurysms. Models extrapolated from existing data have been proposed to guide treatment recommendations. The aim of this study is to assess whether a consensus-based treatment score (UIATS) or rupture rate estimation model (PHASES) can be used to benchmark UK multi-disciplinary team (MDT) practice.

**Methods:**

Prospective data was collected on a consecutive series of all patients with unruptured intracranial aneurysms (UIAs) presenting to a major UK neurovascular centre between 2012 and 2015. The agreement between the UIATS and PHASES scores, and their sensitivity and specificity in predicting the real-world MDT outcome were calculated and compared.

**Results:**

A total of 366 patients (456 aneurysms) were included in the analysis. The agreement between UIATS and MDT recommendation was low (weighted kappa 0.26 [95% CI 0.19, 0.32]); sensitivity and specificity were also low at 36% and 52% respectively. Groups that the MDT allocated to treatment, equipoise or no treatment had significantly different PHASES scores (*p* = 0.004). There was no significant difference between the two scores when predicting patients for whom MDT outcome was to recommend aneurysm treatment, but the UIATS score was superior in predicting patients who received an MDT recommendation of ‘treatment-equipoise’, or ‘not-for-treatment’ (AUC of 0.73 compared to 0.59 for PHASES).

**Conclusions:**

The models studied failed to agree with the consensus view of multi-disciplinary team in a major neurovascular centre. We conclude that decision support tools such as the UIATS and PHASES scores should not be blindly introduced in respective institutions without prior internal validation, as they may not represent the local reality.

## Introduction

The estimated prevalence of unruptured intracranial aneurysms (UIAs) is 3–5% in adults, although diagnosis has become more common with the proliferation of sensitive cross-sectional imaging [27, 29]. Rupture of intracranial aneurysms has an overall mortality of up to 50%, and more than half of the survivors are left with substantial lifestyle restrictions [13, 19]. Treatment of unruptured intracranial aneurysms may also be associated with substantial morbidity; one meta-analysis suggested a case fatality of 1.7% with surgery, moderate morbidity of 5%, and total unfavourable outcome estimate of 6.7% up to 1 year after surgery [14]. Various factors are known to be associated with increased morbidity, including increasing patient age and aneurysm location [28]. Individualised and accurate prediction of UIA rupture risk as well as of treatment morbidity is highly desirable. However, advice must be guided by a limited evidence base combined with clinician acumen. The American Heart Association (AHA) Stroke Council recommendations on treatment of UIAs effectively reflect the complexity of decision-making [25].

The Unruptured Intracranial Aneurysm Treatment Score (UIATS) and the PHASES score are decision support tools designed to guide management of UIAs. The former is a consensus-based scoring strategy based on the recommendations of 69 highly informed individuals [7]; the latter is a 5-year rupture rate estimation synthesizing data drawn from over 8000 patients diagnosed with UIAs [10].

This study examines the level of agreement between either the UIATS or PHASES scores with outcomes from multi-disciplinary team (MDT) review at a high-volume UK tertiary neurovascular centre over a 3-year period.

## Methods

The data that support the findings of this study are available from the corresponding author upon reasonable request. NHS Research Ethics Committee approval was assessed and not required. Patient consent was waived.

### Inclusion and exclusion criteria

A retrospective review of prospectively collected data was carried out on all patients above the age of 18 diagnosed with an UIA and referred to the regional MDT between 2012 and 2015. This unit meets commonly accepted definitions for high treatment volume of cerebral aneurysms [3, 30].

All adult patients with unruptured intracranial aneurysms discussed at the MDT between 2012 and 2015 were included. Fusiform, traumatic and infective aneurysms were excluded. Twenty-nine patients for whom information relating to any of the components UIATS and PHASES were also missing were excluded from the analysis.

The multi-disciplinary team (consisting of two consultant neurosurgeons, one neurovascular fellow, four consultant neuroradiologists, one neurologist and two neurovascular nurse specialists) assess the patients’ medical history and vascular imaging to formulate a management recommendation. Where there is equipoise whether to undertake treatment or as to the modality of treatment to be offered patients is seen by a consultant interventional neuroradiologist and a consultant neurovascular surgeon together. Clinic letters are copied to the patient and printed material as well as online patient information resources are provided. Patients are invited for a second consultation after reflection on that discussion. Nurse specialists provide a further point of contact and the goal is to involve the patient fully in the decision-making process first educating them about the condition and applying a shared decision-making model [20].

### Data collection

Three authors (AKV, JF and JKV) who were not involved in the clinical decision-making for these patients searched the patients’ medical records and prospectively populated departmental database for data relating to each of the components of the UIATS and PHASES scores. Demographic and patient-specific information included age, sex and ethnicity, relevant past medical history and psychological factors relating to the presence of the aneurysm. Clinical and aneurysm-specific information collected consisted of history of previous subarachnoid haemorrhage (SAH), aneurysm multiplicity, aneurysm morphology (lobulation, size, aspect ratio and size ratio [5, 26]) and aneurysm location, as well as the sequelae of the aneurysm, including clinical and radiological mass effect, thrombo-embolic effect, secondary seizures, aneurysm growth or de novo aneurysm formation on serial imaging and contralateral steno-occlusive disease. The UIATS and PHASES scores were calculated according to the methods described in their design [7, 10].

The recommendations were classified into three ordinal groups: ‘for-treatment’, ‘treatment-equipoise’ and ‘not-for-treatment’.

### Statistical analysis

Statistical analysis was undertaken by an independent statistician (Statsconsultancy Ltd.).

#### Comparison of UIATS outcome with MDT outcome

The *overall* agreement between the UIATS outcomes and the MDT outcomes was assessed using the McNemar test, which was used to establish if the UIATS score over- or under-treated relative to the MDT [17]. The agreement between UIATS and the MDT *at the individual aneurysm level* was assessed using the weighted kappa method [15]. This statistic measures the agreement over and above that which would be expected due to chance. The weighted kappa method was preferred to the standard kappa method to allow for the ordinal nature of the decision categories. The statistic is measured on a scale ranging up to a maximum agreement of 1. The sensitivity and specificity of UIATS for the prediction of the MDT decision were also calculated. Sensitivity was calculated by the proportion of cases classified for-treatment by the MDT that were classed as for-treatment by UIATS. Specificity was obtained by calculating the proportion of cases classified not-for-treatment by the MDT that were classed as not-for-treatment by UIATS.

The UIATS outcome was then examined on a continuous scale (in other words, the absolute UIATS score) rather than using the pre-defined ordinal treatment categories. The absolute UIATS scores were found to be normally distributed, and thus, analysis of variance (ANOVA) was used to compare these with the three different MDT recommendations. In addition, ANOVA post hoc tests were performed to compare absolute UIATS scores with pairs of MDT recommendations (for example the composite of ‘treatment’ and ‘equipoise’ recommendations). The *p* values from the post hoc tests were given a Bonferroni adjustment to account for multiple comparisons [6].

#### Comparison of PHASES outcome with MDT outcome

The association between the PHASES score and the MDT outcome was also examined. In contrast to the UIATS scores, the PHASES scores were found not to follow a normal distribution, and therefore, the Kruskal–Wallis test was preferred to compare these with the MDT recommendations. A post hoc comparison was also performed using the Mann–Whitney test, and a Bonferroni correction was again applied.

#### Receiver operating characteristic curves for treat vs. not-treat

The power of each score to predict the MDT outcome was examined by creating a receiver operator characteristic (ROC) curve for the UIATS and PHASES outcomes respectively. As each aneurysm was assessed using both measures, the area under the curves was compared based on correlated ROC curves using the method suggested by DeLong, DeLong and Clarke-Pearson [4]. In order to dichotomize, two different MDT scenarios were analysed. Firstly, aneurysms were classified as either ‘for-treatment’ or alternate outcome (i.e. ‘treatment-equipoise’ or ‘not-for-treatment’), and secondly as ‘not-for-treatment’ or alternate outcome (i.e. ‘for-treatment’ or ‘treatment-equipoise’).

## Results

### Patient characteristics

A total of 456 aneurysms in 366 patients were included in this analysis. The mean age of the patient group was 60 (± 13.9) years, and the majority (82%) had a single aneurysm (13% two aneurysms, 3% three aneurysms and 2% four or more aneurysms). The median maximum diameter of the aneurysms was 6 mm (IQR 4–9 mm). The prevalence of known risk factors for the formation of aneurysms was identified as follows: 3% with previous subarachnoid haemorrhage, 2% with a family history, 26% (current) smokers, 39% hypertensive (established, on treatment) and 3% with adult polycystic kidney disease (see Table 1).

**Table 1:** Patient and aneurysm characteristics

Patient/aneurysm characteristics	Variable	Factor present, number (%)
Risk factors ^(*)^	Previous SAH	10 (3%)
	Fam. history aneurysms	8 (2%)
	Smoking	97 (26%)
	Hypertension	144 (39%)
	APKD	10 (3%)
	IVDU	1 (0.3%)
	Alcohol	8 (2%)
Symptoms ^(*)^	CN deficit	10 (3%)
	Mass effect	16 (4%)
	Thromboembolic event	0 (0%)
	Epilepsy	6 (2%)
Other ^(*)^	Reduced QoL	13 (4%)
	Multiplicity	96 (26%)
Comorbidities ^(*)^	Neurocognitive	7 (2%)
	Coagulopathies	4 (1%)
	Psychiatric diagnosis	6 (2%)
Morphology ^(**)^	Irregularity	26 (6%)
	Size ratio	280 (61%)
Other ^(**)^	Aneurysm growth	13 (3%)
	De novo formation	1 (0.2%)
	Contralateral	0 (0%)
	Complex fistula	101 (22%)

^*^Summaries at the patient level

^**^Summaries at the aneurysm level

*SAH* subarachnoid haemorrhage, *APKD* adult polycystic kidney disease, *IVDU* intra-venous drug use, *CN* cranial nerve, *QoL* quality of life

Of the 319 aneurysms for which the MDT recommended conservative management, patients elected for treatment in 15 (4.7%; 7 surgical, 8 endovascular). In the 73 patients in whom treatment was recommended, only one patient elected to be managed conservatively.

The patient cohort is very similar to that studied in the publications that contribute towards the PHASES analysis (e.g. previous SAH, smoking and hypertension) [10].

### Comparison of UIATS outcome with MDT outcome

The *overall* agreement between the recommendations of the two scores was first examined. The MDT made a recommendation ‘for-treatment’ in 16% of patients, 70% of all patients were classified as ‘not-for-treatment’ by the MDT and in 14% the outcome was ‘treatment-equipoise’. The UIATS score also recommended ‘for-treatment’ in 16% of patients, ‘not-for-treatment’ in 42% of patients and ‘treatment-equipoise’ in 41% of patients. The McNemar test demonstrated that there was a significant difference overall between the two methodologies in terms of outcome. (*p* < 0.001). This difference was almost entirely driven by the ‘treatment-equipoise’ decision at the expense of the ‘not-for-treatment’ recommendation group, in the UIATS cohort.

The relationship between the UIATS and MDT recommendations was then examined at an *individual aneurysm level*. Table 2 cross-tabulates the recommendation of the UIATS method and the MDT outcomes. There was agreement between UIATS and MDT outcome for only 50% of aneurysms (229/456). Surprisingly, however, of those given a ‘for-treatment’ recommendation by UIATS, 43% were given a ‘not-for-treatment’ recommendation by the MDT. A more formal assessment of the correlation between the two methods at the individual aneurysm level, using the weighted kappa score, revealed only fair agreement between the two (weighted kappa 0.26). Sensitivity was also low (36%), suggesting that just over a third of cases given a ‘for-treatment’ outcome by the MDT had the same outcome from UIATS. Specificity was similarly low (52%), suggesting that only half of aneurysms classified as ‘not-for-treatment’ by the MDT were similarly classified by UIATS.

**Table 2:** Cross-tabulation of MDT and UIATS outcomes

MDT outcome	UIATS outcome		
	Not-for-treatment	Treatment-equipoise	For-treatment
Not-for-treatment	167	120	32
Treatment-equipoise	12	36	16
For-treatment	13	34	26

The difference between factors favouring treatment and factors favouring conservative management in the UIATS process (ΔUIATS, the numerical UIATS score) was also examined on a continuous scale rather than categorised by overall outcome. A comparison of the ΔUIATS scores was made between the MDT outcomes. Summaries of the scores in each outcome group are shown in Table 3, along with the overall significance in scores between the three outcome groups. The analysis demonstrated a significant difference in UIATS scores between the MDT outcomes.

**Table 3:** Comparison of ΔUIATS between MDT outcomes

MDT outcome	ΔUIATS, mean ± SD	Overall *p* value
Not-for-treatment	− 3.0 ± 4.4	< 0.001
Treatment-equipoise	0.4 ± 3.4	
For-treatment	1.1 ± 4.7	

**Fig. 1:**
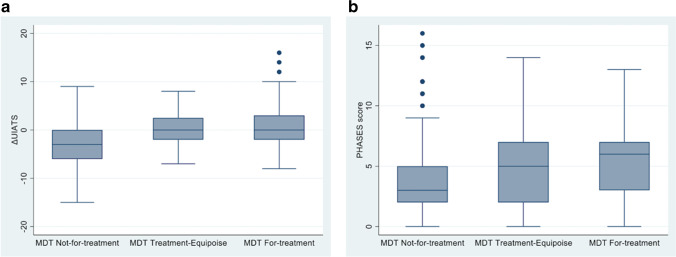
**a** ΔUIATS boxplot for different MDT outcomes. **b** PHASES boxplot for different MDT outcomes

However, post hoc comparisons between pairs of groups suggested significant differences between the MDT ‘not-for-treatment’ outcome and each of the ‘treatment-equipoise’ and ‘for-treatment’ outcomes (*p* < 0.001). There was no difference in scores between the ‘treatment-equipoise’ and ‘for-treatment’ outcomes (*p* = 1.00). Nevertheless, the small differences in ΔUIATS scores between the outcomes suggest that it may be difficult to use ΔUIATS to distinguish between these MDT decisions. A graphical illustration of the scores in the different MDT categories is shown in Fig. 1a.

### Comparison of PHASES outcome with MDT outcome

The PHASES risk-prediction scores for each of the MDT outcome are summarised in Table 4. Due to the skewed distribution of the scores, the median and inter-quartile range are used as the summary measures. Similar to the UIATS score, there was a significant difference in PHASES scores between each MDT outcome. As would be expected, PHASES scores were lowest in the ‘not-for-treatment’ outcome group, and highest in the ‘for-treatment’ outcome group.

**Table 4:** Comparison of PHASES risk-prediction score between MDT outcomes

MDT outcome	PHASES, median [IQR]	Overall *p* value
Not-for-treatment	3 [2, 5]	0.004
Treatment-equipoise	5 [2, 7]	
For-treatment	6 [3, 7]	

Additional Mann–Whitney tests comparing between pairs of outcomes demonstrated no statistically significant difference between adjacent categories (‘not-for-treatment’ vs ‘treatment-equipoise’, *p* = 0.53; ‘treatment-equipoise’ vs. ‘for-treatment’, *p* = 0.48), but a significant difference in PHASES scores between the ‘not-for-treatment’ and ‘for-treatment’ outcomes by the MDT (*p* = 0.003). The results are illustrated graphically in Fig. 1b. The absolute PHASES score therefore was discriminatory for these two MDT outcomes.

### Comparison of receiver operator characteristic curves

Receiver operator characteristic curves were generated for each methodology’s ability to predict each of the three possible MDT outcomes. There are non-significant differences between the areas under each curve (AUC) when predicting a ‘for-treatment’ outcome.

**Fig. 2:**
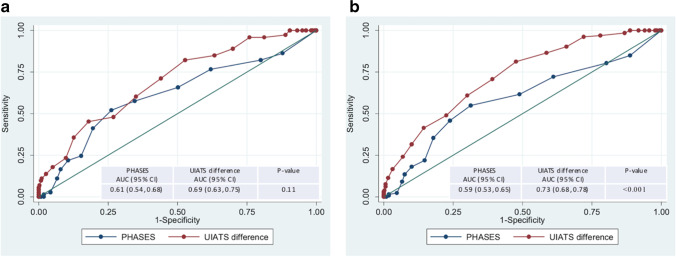
Receiver operating characteristic curves. **a** For PHASES and UIATS prediction of MDT outcomes ‘treatment-equipoise’ or ‘for-treatment’. **b** For PHASES and UIATS prediction of MDT outcome ‘for-treatment’

UIATS and PHASES did significantly differ in their prediction of ‘treatment-equipoise’ and ‘for-treatment’. Here, the UIATS score was superior to the PHASES score, with an AUC of 0.73 compared to 0.59 for PHASES (*p* < 0.001). Graphical illustrations of the results for the two scores are shown in Fig. 2.

## Discussion

Decisions about treatment of unruptured intracranial aneurysms are challenging given the continuing uncertainty as to the natural history of the disease and the risk to benefit balance of prophylactic treatment. Traditionally recommendations have been made by a multi-disciplinary team of professionals combining experience and expertise from a number of specialties and individuals. The UIATS score and to a lesser degree the PHASES score are an attempt to standardize this process. UIATS resulted from a validated, consensus-based strategy (Delphi) to collate parameters considered by a group of 69 highly informed individuals as important in the decision-making process. Using 30 index cases, the authors found a high inter-rater agreement among this group and the score. There are limitations to this technique however and the potential to selectively report consensus in healthcare research has been reported along with methodological precautions required to prevent it [9]. The PHASES methodology is a 5-year rupture estimation score developed by pooling data from the six largest longitudinal studies measuring the rate of aneurysm rupture. It is based on six easily obtainable parameters (aneurysm size and location, hypertension, previous subarachnoid haemorrhage, patient age and geographic location). Two recent studies have attempted to validate the scores independently, either by retrospectively assessing a classifier based on the PHASES score [2] or by externally validating the UIATS score [21]. The PHASES score differs from the UIATS score in that it does not take into account the risk of intervention and focusses on prediction of rupture risk alone. UIATS attempts to balance the risks. It does not adjust for some additional, known risk factors such as familial aneurysms. This simultaneously validates the two scores for the same dataset and against a real-world multi-disciplinary team.

Using various statistical measures, we demonstrated a disagreement between the two scores and the recommendations made at our multi-disciplinary meeting both for the patient and at the individual aneurysm level. In fact, the MDT outcomes agreed with the UIATS recommendations in only 50% of the aneurysms. In some cases, this disparity is striking. For example, the UIATS recommends treatment in 32 of the aneurysms that the MDT recommended conservative management, and the MDT recommends treatment in 13 aneurysms where UIATS pointed towards a conservative approach. In addition, UIATS frequently failed to provide a definitive recommendation for patients; 190 patients in our series (41%) had a UIATS score between − 2 and + 2 (i.e. equipoise). The MDT was able to make a definitive recommendation for 86% of patients.

The reasons for this difference are multiple and challenging to identify. Studies exploring factors affecting the decision of an expert team identified the presence of multiple aneurysms, large aneurysm size, lower comorbidities and younger age and patient fear of rupture as important in this process, and perhaps these should be given more emphasis in treatment scores [1, 11]. The latter is a particularly important factor, given that the members of the MDT will interact with a large number of these patients and are likely to be influenced by a number of factors that are difficult to quantify in these scores, as clinicians are able to interact with individual patients and incorporate their wishes and health beliefs in a joint decision regarding the management of their aneurysm. This interaction does not lend itself to documentation amenable to empirical assessment [8]. Other factors may also account for the difference between the MDT decisions and the recommendations from these scores. It is increasingly demonstrated in the literature that patient psychological factors such as the perception of risk play an important role. Guan et al. emphasise the importance of the distance that the patient lives from the hospital in decision-making, a factor that is likely to play a disproportional role in developing countries [2]. Other authors made the point that a surgeon’s or a centre’s experience also plays into the decision-making locally [3], again not reflected in these scores [4]. More importantly, the same factors taken into account by the scores are given different individual weighting by local experts, highlighting the local reality [5].

Equally pertinent is the issue of external validity. It may well be the case that our population of patients may not be representative of the ‘ideal’ population on which the scores are based. The population covered by our institution is often of lower socio-economic status compared to other areas of London, with slightly lower life expectancy and higher incidence of uncontrolled or undiagnosed severe hypertension and smoking. These factors may well bias the recommended outcome in a standardized score but are taken into account by the astute clinician who has knowledge of the local idiosyncrasies. In addition, our institution surgically clips a higher proportion of aneurysms compared to most other institutions in the country. The modality of treatment is not taken into consideration by the UIATS score, although it is accepted that open surgery and neuro-interventional procedures have different risk profiles and long-term outcomes.

Other studies have also looked at the agreement between these scores and their MDTs. Mateo et al. [16] found that the UIATS and PHASES recommendations do not match those of their MDT, and cite local institutional factors as a potential cause for the disagreement. The National Hospital for Neurology and Neurosurgery in London performed a retrospective audit of 296 patients comparing the recommendations by UIATS with those of the local MDT [23]. They found significant disagreement between the two bodies of expert opinion, and noted that the aneurysm size in particular was a factor that weighted heavily in their local MDT compared to the UIATS score, and that the agreement has increased in more recent years. Ravindra et al. found that the UIATS score recommended over-treatment of the unruptured aneurysms compared to real-world experience in their own practice, reporting on 221 patients [22]. Other recent studies have come to similar conclusions [12, 24]. In the only relevant prospective study, Molenberg et al. found that the UIATS score did not reliably predict the risk of rupture or enlargement of the aneurysms [18]. There is therefore a growing body of literature highlighting the limitations of these decision support tools when it comes to applying them to local settings and questioning their external and internal validity and accuracy in predicting outcome. These studies should serve as a warning to clinicians to avoid relying on these scores blindly for decision-making (Table 5).

**Table 5:** Literature highlighting the limitations of the decision support tools

Study reference	Country	PHASES or UIATS score	Number of patients in the study	Main result	Comments
(*n* = 6)					
Smedley A., Yusupov N., et al. Management of incidental aneurysms: comparison of single centre multi-disciplinary team decision making with the unruptured incidental aneurysm treatment score. Br J Neurosurg 2018;32:526–40	UK	UIATS	296	Disagreement between UIATS and MDT decisions	Aneurysm size a major reason for discrepancy
Ravindra VM, de Havenon A., et al. Validation of the unruptured intracranial aneurysm treatment score: comparison with real-world cerebrovascular practice. *J Neurosurg* 2018; 129(1): 100–106	USA	UIATS	221	UIATS recommendations disagree with those of the treating clinicians	Overall UIATS recommended overtreatment, with large percentage without treatment recommendation
Molenberg R, Aalbers MW, et al. The Unruptured Intracranial Aneurysm Treatment Score as a predictor of aneurysm growth or rupture. *Eur J Neurol* 2020; Online ahead of Print	Netherlands	UIATS	214	Poor performance of UIATS to predict aneurysm growth or rupture	Prospective study following up aneurysms for enlargement or rupture
Stumpo V, Latour K, et al. Retrospective application of UIATS recommendations to a multicenter cohort of ruptured intracranial aneurysms: how it would have oriented the treatment choices?. *World Neurosurg* 2020; S1878-8750(20)32,607–3	Italy	UIATS	146	For 72.6% of the patients, UIATS failed to provide a clear recommendation for treatment	
Hernandez-Duran S, Mielke D., et al. Is the unruptured intracranial aneurysm treatment score (UIATS) sensitive enough to detect aneurysms at risk of rupture? *Neurosurg Rev* 2020; Online ahead of print	Germany	UIATS	212	UIATS shows low sensitivity for detecting aneurysms at risk of rupture	
Bijlenga P, Gondar R., et al. PHASES Score for the management of intracranial aneurysm: a cross-sectional population-based retrospective study. *Stroke* 2017; 48(8): 2105–12	Switzerland	PHASES	841	Progression of PHASES scores between the different groups defined by the authors	Even a low PHASES score is associated with a non-negligible risk of rupture

Our study benefits from a large number of patients and individual aneurysms. It is to our knowledge the largest study to date comparing the PHASES and UIATS scores to local practice. It is also the first study looking at both of these scores simultaneously on the same population of patients. It also benefits from a statistical analysis using a number of different measures to assess agreement. Although the retrospective design of our study fits with our main objective which was to compare the scores’ recommendations with those of our local experts, data are not prospectively sought risk being incomplete, with certain factors being under-reported.

We should be careful when drawing conclusions from our data. The purpose of this work is not to demonstrate superiority of our MDT process over the two decision support tools that we examined. This statement cannot be reliably made without adequate prospectively collected outcome data. It aims instead to highlight that these scores do not correlate well with the MDT decisions, and send a powerful message that internal validation should be considered before adopting them to a particular institution and population. Until then, the UIATS and PHASES scores should probably be regarded as another expert opinion. The study also demonstrates there remains significant uncertainty as to how the majority of UIAs should be managed. This uncertainty needs be communicated to patients as part of shared decision-making.

## Abbreviations

AUC: Areas under each curve
IQR: Interquartile range
MDT: Multi-disciplinary team
PHASES: Population, Hypertension, Age, Size, Earlier subarachnoid haemorrhage, and Site prediction model
UIA: Unruptured intracranial aneurysms
UIATS: Unruptured Intracranial Aneurysm Treatment Score

## References

[1] Alshafai N, Falenchuk O, Cusimano MD (2015). Practises and controversies in the management of asymptomatic aneurysms: results of an international survey. Br J Neurosurg.

[2] Bijlenga P, Gondar R, Schilling S, Morel S, Hirsch S, Cuony J, Corniola M-V, Perren F, Rüfenacht D, Schaller K (2017). PHASES score for the management of intracranial aneurysm: a cross-sectional population-based retrospective study. Stroke.

[3] Connolly ESJ, Rabinstein AA, Carhuapoma JR (2012). Guidelines for the management of aneurysmal subarachnoid hemorrhage: a guideline for healthcare professionals from the American Heart Association/American Stroke Association. Stroke.

[4] DeLong ER, DeLong DM, Clarke-Pearson DL (1988). Comparing the areas under two or more correlated receiver operating characteristic curves: a nonparametric approach. Biometrics.

[5] Dhar S, Tremmel M, Mocco J, Kim M, Yamamoto J, Siddiqui AH, Hopkins LN, Meng H (2008). Morphology parameters for intracranial aneurysm rupture risk assessment. Neurosurgery.

[6] Dunn OJ (1961). Multiple comparisons among means. J Am Stat Assoc.

[7] Etminan N, Brown RDJ, Beseoglu K (2015). The unruptured intracranial aneurysm treatment score: a multidisciplinary consensus. Neurology.

[8] Gillani RL, Podraza KM, Luthra N, Origitano TC, Schneck MJ (2016). Factors influencing the management of unruptured intracranial aneurysms. Cureus.

[9] Grant S, Booth M, Khodyakov D (2018). Lack of preregistered analysis plans allows unacceptable data mining for and selective reporting of consensus in Delphi studies. J Clin Epidemiol.

[10] Greving JP, Wermer MJH, Brown RDJ (2014). Development of the PHASES score for prediction of risk of rupture of intracranial aneurysms: a pooled analysis of six prospective cohort studies. Lancet Neurol.

[11] Guan J, Karsy M, Couldwell WT, Schmidt RH, Taussky P, MacDonald JD, Park MS (2016). Factors influencing management of unruptured intracranial aneurysms: an analysis of 424 consecutive patients. J Neurosurg JNS.

[12] Hernández-Durán S, Mielke D, Rohde V, Malinova V (2020). Is the unruptured intracranial aneurysm treatment score (UIATS) sensitive enough to detect aneurysms at risk of rupture?. Neurosurg Rev.

[13] Hop JW, Rinkel GJE, Algra A, van Gijn J (1997). Case-fatality rates and functional outcome after subarachnoid hemorrhage. Stroke.

[14] Kotowski M, Naggara O, Darsaut TE, Nolet S, Gevry G, Kouznetsov E, Raymond J (2013). Safety and occlusion rates of surgical treatment of unruptured intracranial aneurysms: a systematic review and meta-analysis of the literature from 1990 to 2011. J Neurol Neurosurg Psychiatry.

[15] Landis J, Koch G (1997). The measurement of observer agreement for categorical data. Biometrics.

[16] Mateo O, Valtueña M, Valés M, García-Leal R, del Valle M, Fortea F, Castro E (2018). Controversies on treatment of unruptured intracranial aneurysms. Value of UIATS and PHASES scores in a daily practice in a Spanish population. Interdiscip Neurosurg.

[17] McNemar Q (1947). Note on the sampling error of the difference between correlated proportions or percentages. Psychometrika.

[18] Molenberg R, Aalbers MW, Mazuri A, Luijckx GJ, Metzemaekers JDM, Groen RJM, Uyttenboogaart M, van Dijk JMC (2020). The unruptured intracranial aneurysm treatment score as a predictor of aneurysm growth or rupture. Eur J Neurol.

[19] Molyneux AJ, Kerr RS, Yu L-M, Clarke M, Sneade M, Yarnold JA, Sandercock P (2005). International subarachnoid aneurysm trial (ISAT) of neurosurgical clipping versus endovascular coiling in 2143 patients with ruptured intracranial aneurysms: a randomised comparison of effects on survival, dependency, seizures, rebleeding, subgroups, and aneurysm occlusion. Lancet.

[20] National Institute for Health and Care Excellence (2015) Shared decision making collaborative a consensus statement. https://www.nice.org.uk/Media/Default/About/what-we-do/SDM-consensus-statement.pdf

[21] Ravindra VM, de Havenon A, Gooldy TC, Scoville J, Guan J, Couldwell WT, Taussky P, MacDonald JD, Schmidt RH, Park MS (2017) Validation of the unruptured intracranial aneurysm treatment score: comparison with real-world cerebrovascular practice. J Neurosurg 1–7 10.3171/2017.4.JNS17548 28984518

[22] Ravindra VM, de Havenon A, Gooldy TC, Scoville J, Guan J, Couldwell WT, Taussky P, MacDonald JD, Schmidt RH, Park MS (2018). Validation of the unruptured intracranial aneurysm treatment score: comparison with real-world cerebrovascular practice. J Neurosurg.

[23] Smedley A, Yusupov N, Almousa A, Solbach T, Toma AK, Grieve JP (2018). Management of incidental aneurysms: comparison of single centre multi-disciplinary team decision making with the unruptured incidental aneurysm treatment score. Br J Neurosurg.

[24] Stumpo V, Latour K, Trevisi G, Valente I, D’Arrigo S, Mangiola A, Olivi A, Sturiale CL (2020). Retrospective application of UIATS recommendations to a multicenter cohort of ruptured intracranial aneurysms: how it would have oriented the treatment choices?. World Neurosurg.

[25] Thompson BG, Brown RDJ, Amin-Hanjani S (2015). Guidelines for the management of patients with unruptured intracranial aneurysms: a guideline for healthcare professionals from the American Heart Association/American Stroke Association. Stroke.

[26] Ujiie H, Tachi H, Hiramatsu O, Hazel AL, Matsumoto T, Ogasawara Y, Nakajima H, Hori T, Takakura K, Kajiya F (1999). Effects of size and shape (aspect ratio) on the hemodynamics of saccular aneurysms: a possible index for surgical treatment of intracranial aneurysms. Neurosurgery.

[27] Vlak MH, Algra A, Brandenburg R, Rinkel GJ (2011). Prevalence of unruptured intracranial aneurysms, with emphasis on sex, age, comorbidity, country, and time period: a systematic review and meta-analysis. Lancet Neurol.

[28] Wiebers DO, Whisnant JP, Huston J (2003). Unruptured intracranial aneurysms: natural history, clinical outcome, and risks of surgical and endovascular treatment. Lancet (London, England).

[29] Wiebers DO, Whisnant JP, Sundt TM, O’Fallon WM (1987). The significance of unruptured intracranial saccular aneurysms. J Neurosurg.

[30] Zacharia BE, Bruce SS, Carpenter AM (2014). Variability in outcome after elective cerebral aneurysm repair in high-volume academic medical centers. Stroke.

